# The mediating role of behavioural and socio-structural factors on the association between household wealth and childhood malaria in Ghana

**DOI:** 10.1186/s12936-024-05204-6

**Published:** 2024-12-13

**Authors:** Theresa Habermann, Solomon T. Wafula, Jürgen May, Eva Lorenz, Dewi Ismajani Puradiredja

**Affiliations:** 1 Department of Infectious Disease Epidemiology, Bernhard Nocht Institute for Tropical Medicine, Hamburg, Germany; 2 Department of Disease Control and Environmental Health, School of Public Health, Makerere University, Kampala, Uganda; 3 German Center for Infection Research (DZIF), Partner Site Hamburg-Borstel-Lübeck-Riems, Hamburg, Germany; 4 Department of Tropical Medicine, University Medical Center Hamburg-Eppendorf (UKE), Hamburg, Germany

**Keywords:** Malaria, Behavioural and socioeconomic factors, Mediation analysis, Children, Ghana

## Abstract

**Background:**

Children under five continue to bear a disproportionate burden of malaria morbidity and mortality in endemic countries. While the link between socioeconomic position (SEP) and malaria is well established, the causal pathways remain poorly understood, hindering the design and implementation of more targeted structural interventions. This study examines the association between SEP and malaria among children in Ghana and explores the potential mediating role of behavioural and socio-structural factors.

**Methods:**

Data from the Ghana Demographic and Health Survey (DHS) 2022 were analysed. As part of the survey, children were tested for malaria using a rapid diagnostic test (RDT), and SEP was measured using a household asset-based wealth index. Mediation analysis (MA) using a regression-based approach was performed to assess mediated effects between SEP and malaria in children under five in Ghana through housing quality, educational attainment (EA), long-lasting insecticidal net (LLIN) use, indoor residual spraying (IRS), and healthcare-seeking behaviour (HSB). Reported are the total natural indirect effects (TNIEs) and the proportion mediated (PM).

**Results:**

Of the 3,884 children included in the survey, 19.4% (757) had malaria. Belonging to a household with high SEP was associated with a 43% lower risk of malaria (Prevalence Ratio, PR = 0.57; 95% Confidence Interval, CI 0.46–0.71). Regarding indirect (mediated) effects, maternal EA of secondary school or higher (OR = 0.68; 95% CI 0.60–0.77; PM = 17.5%), improved housing (OR = 0.80; 95% CI 0.68–0.91, PM = 9.2%), LLIN use (OR = 0.95; 95% CI 0.90–0.99, PM = 2.1%) partially mediated the association between SEP and malaria. The combined effect of all three mediators was higher than those in a single mediator or two sequential mediators (with EA as the initial mediator) (OR = 0.58; 95% CI 0.51–0.68, PM = 25.7%). No evidence of mediation was observed for HSB and IRS.

**Conclusion:**

We found evidence of mediation by EA, housing, LLIN use and IRS, suggesting that current biomedical and behavioural malaria control efforts could be complemented with structural interventions, such as improved housing and education. Future studies that test the effect of different or joint effects of multiple mediators based on prospective designs are recommended to strengthen the evidence.

**Supplementary Information:**

The online version contains supplementary material available at 10.1186/s12936-024-05204-6.

## Background

Malaria in children remains a major public health concern in sub-Saharan Africa (SSA), particularly in Ghana. In areas where malaria is endemic, children of less educated mothers, from low socioeconomic households, and with less frequent use of long-lasting insecticidal nets (LLINs) are more likely to be infected [1, 2]. Previous studies have demonstrated an association between poverty and malaria, yet it remains challenging to understand the magnitude and directionality of this relationship [3, 4]. Wealth can be protective against malaria through multiple potential mechanisms. It can afford individuals greater access to healthcare services, enable the acquisition and use of LLINs, foster proactive treatment-seeking behaviours (HSB), improve housing and neighbourhood environmental conditions, and ensure better nutrition. These factors collectively contribute to a robust defense against malaria [5–7]. Despite this knowledge, the specific pathways through which household wealth mitigates malaria risk remain underexplored. Traditional methods have fallen short in accurately estimating the indirect effects (mediated effects) and in accounting for the interactions between exposure and potential mediators [8].

Mediation analysis has the potential to enhance the understanding of the apparent linkages between wealth and malaria risk. This analytical approach can illuminate how various conditions associated with wealth influence malaria risk and to what extent these conditions mediate associations between socioeconomic positioning (SEP) and malaria. Current advances enable assessment of the mediating effect of variables beyond the product and difference methods using the counterfactual approach with stringent assumptions, and sensitivity analyses to address these assumptions and to improve the interpretation of findings [9]. The application of mediation analysis can be highly informative in terms of more effective interventions, enabling a more targeted approach that focuses on the most influential pathways [10]. While such insights are valuable for policy and practice, they have not yet been extensively applied in previous research [6, 8, 11]. Building on scarce literature that seeks to understand the mechanisms through which poverty affects malaria [6, 11], this study aimed to estimate the contribution of potential mediators on the pathway between household-level SEP and malaria among children in Ghana from a large, nationwide survey.

## Methods

### Study design

This study utilized data from the Ghana Demographic and Health Survey (DHS) 2022. The DHSs are typically conducted during dry seasons and employ a two-stage stratified-cluster sampling design based on the sampling frame of the population and housing census, stratified by urban/rural areas within the 16 geographic regions of Ghana [12]. Data collection for the Ghana DHS 2022 was conducted between October 2022 and January 2023. Following this, DHS generated several datasets, including a household recode file, (HR), an individual (mother) recode file, a children file, and a member recode file. For this analysis, we merged the modified HR file with the children’s file which contains information on malaria rapid diagnostic test results. However, we limited the sample to 3,884 children who had a valid malaria test result (RDT) and with corresponding matching identifiers with children and household files.

This study was informed by a conceptual framework, which guided the construction of an asset-based wealth index to depict households’ SEP and the subsequent regression and mediation analyses.

### Conceptual framework

**Fig. 1:**
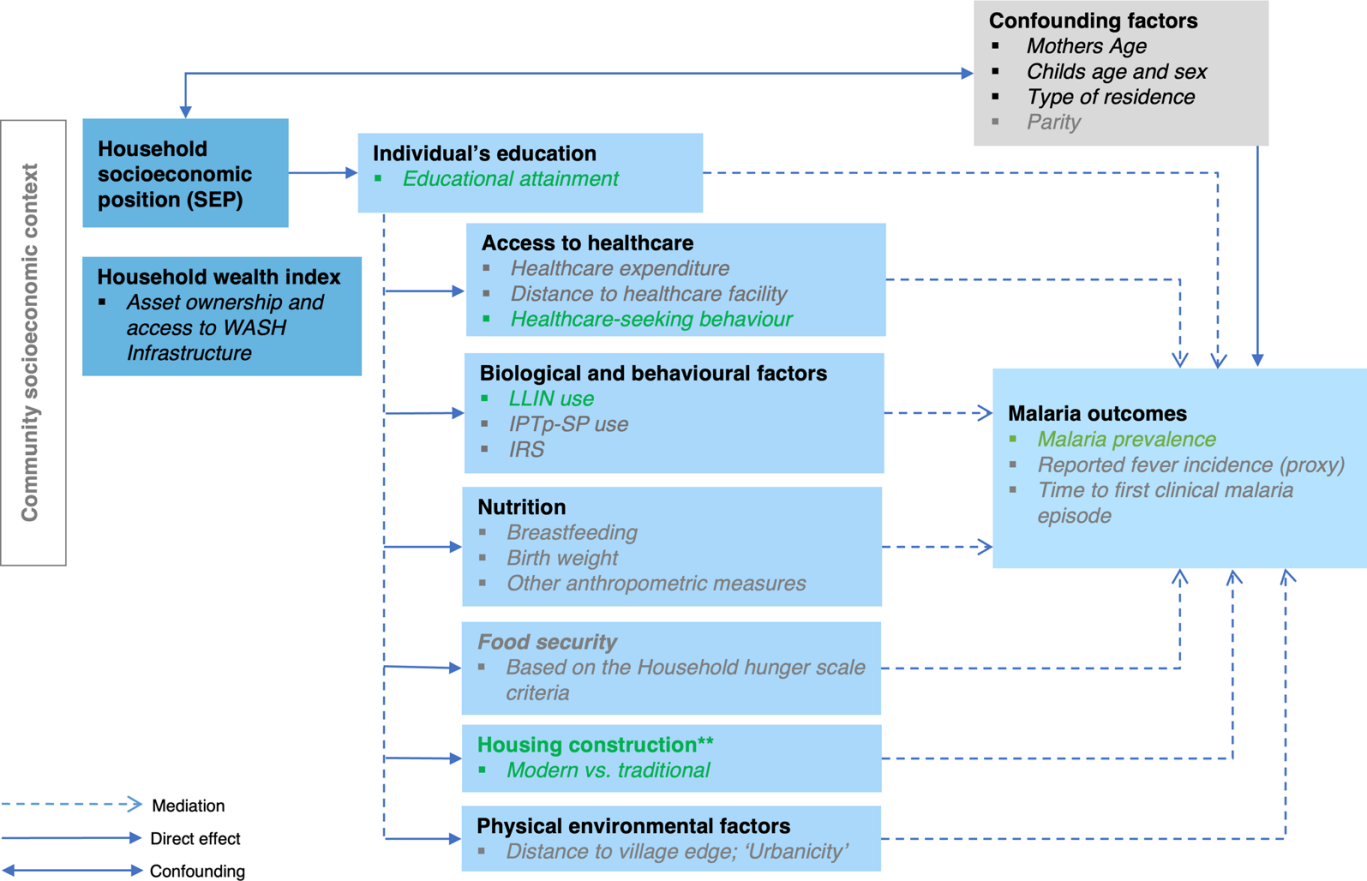
Conceptual framework

***Notes:*** The conceptual framework aims to depict a comprehensive picture of the association between household SEP and malaria in children under 5 years of age in Ghana. While a bi-directional association between malaria and poverty is well-recognized [13], this study focuses on the direction assuming that having a lower SEP, is associated with higher odds of malaria especially in children [14, 15]. This socioeconomic disadvantage can lead to restricted access to healthcare facilities i.e., fewer visits [16], lower quality of housing [17, 18], possibly less knowledge and use about LLINs to prevent malaria infection [2, 19] and lower likelihood to perform indoor residual spraying [20]. Higher maternal educational attainment is associated with a reduced risk of malaria infection in their children because they are more likely to have a higher income and have improved access to preventive actions and healthcare services [8, 21]. Possible additional mediators, shaded in grey, are not provided within DHS 2022 data (Adapted from Tusting, et al., 2016) [6].

We developed a conceptual framework that describes the hypothesized indirect effects between household-level poverty (described using SEP) and malaria (measured using RDTs) through behavioural and socio-structural factors, such as highest maternal EA, housing quality, usage of LLINs, IRS and healthcare-seeking behaviour (HSB) as possible mediators (Fig. 1). Education is the preceding mediator hypothesized to influence other mediators. Malaria is assessed in a subset of children under 5 whether they have symptoms suggestive of malaria or not.

### Variables and measurement

**SEP:** The exposure was household SEP, generated using principal component analysis (PCA) on household assets and household conditions [22]. The assets included in the PCA were electricity, radio, television, refrigerator, motorcycle, car, phone, bank account, cabinet, and household size, all of which were hypothesized to influence wealth in the same direction in both rural and urban settings. All assets were dichotomous except household size which was a discrete variable. In the PCA, we considered a mixed correlation structure and orthogonal varimax rotation to maximize loadings on the principal components (PC). The resulting scores from the first PC were used to generate the SEP variable in line with validated instructions by Rutstein (2015) [23]. The scores were divided into three categories: low SEP (the lowest 40% of the scores), middle SEP (scores from 41 to 80th percentile) and high SEP (the top 20% of the scores) to ease interpretation [24, 25]. We excluded housing variables from this PCA as housing was one of the potential mediators assessed for the association between SEP and malaria.

**Malaria:** The study outcome was malaria, which was determined based on rapid diagnostic test (RDT) results. DHS used *SD Bioline Antigen* RDT to test for the parasite *Plasmodium falciparum* using a drop of blood-based on the principle of antigen–antibody reaction. This was a binary indicator variable with the following options: Positive” (coded 1) or “Negative” (coded 0). Observations with other options (< 2.5%) such as “not available”, “refused”, or “others” were excluded from this analysis.

**Potential mediators:** Informed by prior research [6, 8], we considered the highest maternal educational attainment (EA), housing quality, LLIN use (for children), indoor residual spraying and HSB as potential mediators for the association between household SEP and malaria. These mediators were measured as follows.

- **Maternal educational attainment (EA)**: Initially categorized as “no education, “incomplete primary”, complete primary”, “incomplete secondary”, complete secondary and “higher level”. Due to the few observations in some categories [e.g. incomplete primary 3.1% (121), and education higher than secondary 7.1% (279)], we consolidated EA into two categories: Primary and lower (coded 0) which includes “no education”, “incomplete primary”, and complete primary”; and Secondary or higher (coded 1) which includes “incomplete secondary”, complete secondary and “higher level”.
- **Housing quality:** We used the DHS definitions of finished, rudimentary, and natural materials to classify the type of wall, roof, and floor materials of main dwelling units [26]. We then created a binary variable comparing “modern” (all materials for the roof, floor, and walls are finished materials) with “traditional” housing (at least one of the materials is rudimentary or natural), coded as 0 and 1 respectively.
- **LLIN use:** Defined as a child under 5 years sleeping in the long-lasting insecticidal net (LLIN) the night before the survey [27]. This was reported by parents as “Yes” (coded 1) or “No” (coded 0).
- **Indoor residual spraying (IRS)**: This was a binary variable which assessed whether IRS had been conducted at the household in the previous 6 months with responses “Yes” (coded 1) or “No” (coded 0).
- **Healthcare-seeking behaviour (HSB)**: We used the number of times a mother visited a health facility in the last 6 months as a proxy for HSB. Mothers were categorized according to the frequency with which they had visited a healthcare facility in the last 6 months (no health visit vs. at least one visit).
- **Covariates:** We included variables known or hypothesized to be associated with malaria and/or SEP. These variables include the children’s age (in complete months) and sex, and the mothers' age (in complete years) [28]. Beyond individual characteristics, we included whether a child resided in a rural or urban setting to control for unmeasured variability at the community level. All variables and their intricate associations are shown in the framework (Fig. 1).

### Statistical analyses

Analyses were conducted at three levels. Firstly, we presented categorical demographic data (of households, mothers and children) as frequencies and percentages both overall and stratified by type of malaria RDT test result. Continuous variables such as the age of the children and the mothers were summarized using mean and standard deviations. Pairwise comparisons for the distribution of malarial infection by participant characteristics, SEP and the mediators were conducted and p-values (corrected for multiple testing) have been provided (Table 1).

**Table 1:** Distribution of participants by malarial infection status

Characteristic	Total, N = 3,884^1^	No infection, N = 3,127^1^	Infection, N = 757^1^	p-value^2^
Age of the child (in months)	30.7 (15.4)	29.8 (15.4)	34.3 (14.9)	< 0.001
Sex of the child				0.848
Male	1,967 (50.6%)	1,586 (80.6%)	381 (19.4%)	
Female	1,917 (49.4%)	1,541 (80.4%)	376 (19.6%)	
Residence				< 0.001
Urban	1,612 (41.5%)	1,460 (90.6%)	152 (9.4%)	
Rural	2,272 (58.5%)	1,667 (73.4%)	605 (26.6%)	
Mother`s age (in years)	31.0 (6.9)	31.1 (6.8)	30.7 (7.2)	0.201
Socioeconomic position (SEP)				< 0.001
Low	1,707 (43.9%)	1,219 (71.4%)	488 (28.6%)	
Middle	1,528 (39.3%)	1,294 (84.7%)	234 (15.3%)	
High	649 (16.7%)	614 (94.9%)	35 (5.4%)	
Maternal education (EA)				< 0.001
Primary school or lower	1,846 (47.5%)	1,363 (73.8%)	483 (26.2%)	
Secondary School or higher	2,038 (52.5%)	1,764 (86.6%)	274 (13.4%)	
Slept under a LLIN the previous night				< 0.001
No	1,709 (44.0%)	1,422 (83.2%)	287 (16.8%)	
Yes	2,175 (56.0%)	1,705 (78.4%)	470 (21.6%)	
Lives in a modern house				< 0.001
No	1,434 (36.9%)	1,066 (74.3%)	368 (25.7%)	
Yes	2,450 (63.1%)	2,061 (84.1%)	389 (15.9%)	
IRS in the past 12 months				< 0.001
No	3,139 (81.2%)	2,566 (81.7%)	573 (18.3%)	
Yes	728 (18.8%)	544 (74.7%)	184 (25.3%)	
Healthcare-seeking behaviour (HSB)				0.726
No health visit	2,673 (68.8%)	2,147 (80.3%)	526 (19.7%)	
At least one visit	1,211 (31.2%)	980 (80.9%)	231 (19.1%)	

*EA* Educational Attainment, *HSB* Healthcare-seeking behaviour, *LLIN* Long-lasting insecticidal net, *IRS* Indoor residual spraying, *SEP* socioeconomic position

^1^Mean (SD); n (%)

^2^Welch Two Sample t-test; Pearson’s Chi-squared test; Benjamín & Hochberg correction for multiple testing

Next, we modelled the association between SEP and malaria using multivariable modified Poisson regression, adjusting for children’s age, gender, residence type, maternal age, education, IRS use, LLIN use and HSB and estimates are presented as Prevalence Ratios (PRs) and 95% confidence intervals (CIs). The modified Poisson is more appropriate when the outcome is common (prevalence > 10%) since odds ratios from logistic regression can’t accurately approximate relative risk [29]. Ordinary Poisson model would be incorrectly specified with respect to the outcome, providing invalid standard errors, however modified Poisson allowed standard errors to be estimated robustly. For interpretation, the prevalence ratio measures the proportion of participants with an outcome (e.g. malaria) in the exposed group (e.g. high SEP) compared to the unexposed group (e.g. low SEP). A PR of 0.5 indicates that the prevalence of malaria is 50% lower among those exposed (in higher SEP) compared to those who are not exposed (those in low SEP).

Lastly, we performed mediation analyses using a regression-based approach [30] to assess the mediating role of individual factors: maternal EA, LLIN use, IRS, housing quality and HSB on the association between SEP and malaria. Following the single mediator analyses, we performed four sequential mediation analyses with maternal EA as the initial mediator and EA, LLIN use, IRS, housing quality and HSB as the second mediators, respectively. Thereafter, all significant mediators from single models were combined for joint effect in multiple mediator analyses. The analyses conducted using the R package, CMAverse [31], provided the total effect estimates (TE), total natural direct effect (TNDE) and total natural indirect effect (TNIE) on the odds ratio scale as well as the proportion mediated (PM). For mediation analysis, TNIE and PM are of most interest for interpretation as they quantify the magnitude of the mediated effects. However, we also report the marginal direct effects. The effect of each of the mediators was assessed separately and those with significant mediation effects in single mediator models were then added into a multiple mediator model to assess their combined effect. We assessed the robustness of the mediation estimates to unmeasured confounding and reported the respective E-values (on the risk ratio scale) and corresponding confidence intervals closest to the null. The E-value which assesses the impact of unmeasured confounding, ranges from 1 to + ∝ . Larger E-values indicate a stronger association that is less likely to be nullified by unmeasured confounders, while lower E-values (especially when close to 1) signal vulnerability to such confounding. The results from two of the criteria for mediation according to Baron and Kenny [32] are shown in Supplementary file 1.

### Ethical considerations

This study utilized secondary data from which all personal identifiers had been removed. These data are accessible to authorized individuals via online DHS repositories (www.dhsprogram.com). The DHS Program’s procedures and questionnaires have undergone a review process and received approval from the ICF International Institutional Review Board (IRB). Participation in DHS surveys is based on voluntary written informed consent.

## Results

### Characteristics of the study population

The study included a total of 3,884 children under 5 years, with a mean age of 30.7 months (SD = 15.4). Malaria prevalence was 19.4% (757 participants). More than half, 58.5% (2,272) of the participants lived in rural areas, and 26.6% (605) of these had malarial infection, compared to only 9.4% (152) out of 1,612 in urban areas.

In the low SEP group, 28.6% (488) had a malarial infection, whereas in the high SEP group, 5.4% (35) had malarial infection. Half of the children, 52.5% (2,038), had mothers who had attained secondary school education or higher. While 13.4% (274) of children born to mothers with secondary school education or higher tested positive for malaria, 26.2% (483) tested positive among those whose mothers had primary education or lower.

The largest proportion of children, 63.1% (2,450) lived in modern housing. Among those in modern houses, 15.9% (389) had a malarial infection, as compared to 25.7% (368) in traditional houses (Table 1).

### Association between socioeconomic position and malarial infection

In the adjusted regression models, belonging to middle (PR = 0.72, 95% CI 0.60–0.85) and high socioeconomic strata (PR = 0.35, 95% CI 0.24–0.50) were associated with 28 and 65% less likelihood of testing positive for malaria among children, respectively. Having a mother with secondary school education or higher was protective against malarial infection in children (PR = 0.73, 95% CI 0.62–0.86) while rural residence was associated with a nearly twofold increased likelihood of testing positive for malaria (PR = 1.90, 95% CI 1.56–2.31) compared to residing in urban settings (Table 2).

**Table 2:** Association between SEP determinants and malarial infection

Characteristic	PR^1^	95% CI^1^	p-value^2^
Age of the child	1.02	1.01–1.02	< 0.001
Mother`s age	0.98	0.97–0.99	0.009
Sex of the child			
Male	1		
Female	1.01	0.88–1.17	0.854
Residence			
Urban	1		
Rural	1.90	1.56–2.31	< 0.001
Socioeconomic position			
Low	1		
Middle	0.72	0.60–0.85	< 0.001
High	0.35	0.24–0.50	< 0.001
Maternal education (EA)			
Primary school or lower	1		
Secondary school or higher	0.73	0.62–0.86	< 0.001
Slept in a LLIN the previous night			
No	1		
Yes	1.05	0.90–1.22	0.653
Lives in a modern house			
No	1		
Yes	0.96	0.82–1.12	0.677
IRS in the past 12 months			
No	1		
Yes	1.11	0.93–1.31	0.389
Healthcare-seeking behaviour (HSB)			
No health visit	1		
At least one visit	1.07	0.92–1.25	0.509

*EA* Educational Attainment, *HSB* Healthcare-seeking behaviour, *LLIN* Long-lasting insecticidal net, *IRS* Indoor residual spraying, *SEP* socioeconomic position

^1^*PR* Prevalence Ratio, *CI* Confidence Interval

^2^Benjamini & Hochberg correction for multiple testing

### Mediation analysis

#### Effect decomposition of natural effect models with mediators

The single mediator analyses show that housing quality, maternal EA, and LLIN use partially mediated 9.2, 17.5 and 2.1% of the effect of SEP on malarial infection, respectively. Considering sequential two mediators with EA as the initial mediators, the pathway EA and housing explained 23.9%, followed by EA + LLIN use (19.8%), EA + HSB (17.5%) and lastly through EA + IRS (17.3%).

The joint effect of multiple mediator candidates (EA, Housing quality and LLIN use) explained the highest mediated effect of SEP (25.7%) on malarial infection. The indirect effect through multiple mediators was more robust to unmeasured confounding than through individual mediators as seen by the E-values. This suggests that a relatively small confounding effect could potentially explain the observed individual mediator estimates. Check the indirect effects and E-values in Table 3.

**Table 3:** Total Effect (TE), TNDEs, and TNIE for the association between SEP and malarial infection with different mediators

	Total (N = 3,884)	
Mediators	Estimate (95% CI)	E-Values for unmeasured confounding, RR [CI]
ASingle (individual) mediators		
EA (Secondary or higher)^1,*^		
Total effect (of SEP)	0.27 (0.23–0.33)	6.73 (5.46)
TNDE	0.31 (0.28–0.32)	5.86 (5.64)
TNIE	0.68 (0.60–0.77)	**2.30 (1.92)**
Proportion mediated	**17.5%**	
Housing quality^1^		
TNDE	0.28 (0.23–0.33)	6.71 (5.46)
TNIE	0.80 (0.68–0.91)	**1.79 (1.43)**
Proportion mediated	**9.2%**	
HSB (At least one visit in last 6 month)		
TNDE	0.27 (0.25–0.29)	6.65 (6.42)
TNIE	0.99 (0.97–1.01)	1.13 (1.00)
Proportion mediated	0.4%	
LLIN use^1^		
TNDE	0.29 (0.25–0.36)	6.70 (5.37)
TNIE	0.94 (0.90–0.99)	1.29 (1.01)
Proportion mediated	**2.1%**	
IRS in the last 12 months^1^		
TNDE	0.29 (0.26–0.31)	6.32 (6.00)
TNIE	0.98 (0.96–1.04)	1.15 (1.00)
Proportion mediated	0.6% (NS)	
Sequential mediators (EA as initial mediator)		
EA + Housing quality		
TNDE	0.30 (0.23–0.37)	6.02 (4.82)
TNIE	0.60 (0.53–0.72)	2.70 (2.14)
Proportion mediated	**23.9%**	
EA + HSB		
TNDE	0.31 (0.24–0.35)	6.00 (5.23)
TNIE	0.68 (0.63–0.77)	2.31 (1.93)
Proportion mediated	**17.5%**	
EA + LLIN use ^1^		
TNDE	0.31 (0.24–0.37)	5.93 (4.90)
TNIE	0.65 (0.60–0.73)	2.44 (2.07)
Proportion mediated	**19.8%**	
EA + IRS in the last 12 months^1^		
TNDE	0.32 (0.25–0.37)	5.67 (4.81)
TNIE	0.69 (0.63–0.77)	2.27 (1.92)
Proportion mediated	**17.3%**	
Joint effect through multiple mediators (EA, LLIN use, and Housing quality)^*^		
TNDE	0.30 (0.24–0.39)	6.10 (4.62)
TNIE	0.58 (0.51–0.68)	2.82 (2.32)
Proportion mediated	**25.7%**	

Estimates are presented as odds ratio (95% confidence interval) unless otherwise indicated. Effect estimates with statistical significance are labelled in bold. *All models adjusted for children’s age, sex and age of the mother and residence*

The total effect estimate with its 95% confidence interval was slightly different for each mediation analysis. To reduce overlap, we listed the value common in the model for educational attainment

*EA* Educational Attainment; *HSB* Healthcare-seeking behaviour; *LLIN* Long-lasting insecticidal net; *IRS* Indoor Residual Spraying; *NS* Non-significant; SEP socioeconomic position, *TE* Total effect estimates, *TNDE* Total natural direct effect, *TNIE* Total natural indirect effect, *RR* Risk Ratio

^*^Mediator-exposure interactions considered

The mediator of EA was modelled as “Secondary or higher” vs “primary or lower”

The mediator of housing quality was modelled as high vs low

The mediators of LLIN use and IRS were modelled as yes vs No

The mediator of HSB was modelled as “making at least one health facility visit in the last 6 months” vs “no health facility visits in the last 6 months”

^1^Mediators considered in the multiple mediator model

## Discussion

This study assessed the mediating role of behavioural and socio-structural factors on the pathway between SEP and malaria infection among children in the 2022 Ghana DHS. In this analysis, malarial infection prevalence stood at 19.4% which is consistent with estimates ranging from 18 to 31% in recent studies in Ghana [33–35]. The study on which this analysis is based utilized RDTs to assess malarial infection. RDTs perform better than microscopy due to their ability to detect antigens (and not parasites) and diagnose malaria in people with low parasitaemia [36]. Additionally, RDTs are inexpensive and easier to use in field surveys as they require little technical knowledge [37].

In this study, we found an association between household SEP and malarial infection in line with previous studies [6, 8]. Our findings indicate that children from high SEP households are at lower risk of malaria, confirming findings from Degarege et al*.* [11]. Further, our findings show evidence of the mediating role of higher maternal EA, housing quality (modern housing), and LLIN use, but not of IRS and HSB. While maternal EA mediated the association between SEP and malaria, the indirect effects were only moderately robust to unmeasured confounding variables. High maternal EA has been shown to have a positive impact on a range of health-related outcomes [19, 38, 39]. For instance, high maternal EA has been found to associated with lower risk of malaria infections. This is because EA predicts greater bednet use, and greater social networking, which can facilitate better health care seeking and potentially lower disease infection rates [21, 40]. This study posits that maternal EA, particularly secondary school education or higher, partially mediates the relationship between SEP and malarial infection among children. Higher educational status may not only be correlated with increased awareness of the disease, and the implementation of malaria preventive measures but also with enhanced early symptom recognition and timely healthcare-seeking behaviour [41]. Health education campaigns should therefore focus on mothers with low EA so that they are empowered to make more informed choices that protect their children from malaria. In light of these findings, the study advocates for the promotion of educational advancement as a long-term socio-structural intervention within the broader strategy for malaria control and elimination.

We also found that LLIN use explained 2.5% of the effect of SEP on malaria. While the proportions explained are small, they confirm the protective effectiveness of LLINs against malaria and the influence of SEP on their usage, as previously highlighted [42–44]. In Ghana, universal coverage of LLINs has not yet been achieved [45] and government programmes need to prioritize the distribution of LLINs to low SEP households. The low E-values associated with the mediated effects suggest vulnerability to unmeasured confounding, which means that findings should be interpreted with caution. Nonetheless, even a modest mediated effect suggests that improving LLIN coverage and eventual use could help reduce malaria risk and also reduce disparities in SEP.

Our findings show that living in modern housing was protective against malarial infection and partially mediates the effect of SEP on malaria. As per Baron and Kenny’s criteria [32], higher SEP was associated with a greater likelihood of residing in modern housing, which in turn was associated with a lower risk of malaria. The shown protective effect of modern housing material on malarial infection is further congruent with findings from a multi-national survey by Tusting et al*.* [46] as well as cross-sectional studies in Ghana [47] and Ethiopia[48], as well as cohort studies involving children in Uganda [49, 50]. These findings all suggest a positive impact of modern housing materials, closed eaves and indoor residual spraying in reducing the risk of malaria infection and parasite prevalence. Improved housing is crucial as it limits the entrance of mosquito vectors, ultimately reducing indoor mosquito biting rates [17, 18]. Entry point screening and sealing wall holes are cost-effective strategies that can reduce mosquito entry [51]. Well-designed housing modifications can help reduce the need for LLINs and IRS and hence may prove cost-effective in the long term [52]. It is important to note that housing modifications also have limitations as they do not prevent mosquito bites that occur outside the house, which have increased significantly due to the shifting vector behaviour with more biting occurring in the evening and mornings [53]. Housing strategies should be deployed in an integrated manner along with other behavioural, environmental and structural interventions. For example, spatial repellants could be important components in the malaria control toolkit.

Multiple sequential mediator analyses revealed that the combined effect of EA and any of the other mediators except IRS explained a greater proportion of the effect of SEP on reducing the risk of malaria than when each mediator was considered separately. This suggests that a multifaceted approach, addressing several mediators simultaneously, could substantially reduce malaria incidence rates including those associated with socioeconomic disparities. A recent review [54] highlighted the superior effect of deploying multiple malaria prevention methods as compared to only a single method. Such evidence is instrumental in shaping informed policies and programming for comprehensive malaria control in endemic settings. Since one-fourth of the effect of SEP has been explained by the mediators considered, future studies should explore the contribution of additional pathways.

## Strengths and limitations of the study

Our study applied a counterfactual approach to mediation analysis, shedding light on the intricate underlying pathways linking SEP and malarial infection. Furthermore, we decided to implement a three-step approach to mediation analysis: single analyses, sequential, and combined, which better represented our conceptual framework. However, current methods are not well-suited to handle more than two sequential mediators. Therefore, joint effects in the combined model (at least three mediators) assume the mediators are not sequential, which should be interpreted with that knowledge in mind. Secondly, the cross-sectional design of the study limits our ability to make causal inferences. Thirdly, we also had limited information on additional confounders needed to control mediator-exposure confounding. Nevertheless, we performed sensitivity analyses for unmeasured confounding and provided E-values to guide interpretation. Despite these methodological challenges, the investigation of mediators is a significant step towards identifying potential intervention pathways.

## Conclusion

This study confirmed a high prevalence of malaria among children under the age of five in Ghana and found that a high risk of malaria was associated with lower household SEP. This effect of SEP was partially mediated through maternal EA, housing quality, use of LLINs and IRS. We recommend increased more targeted investments in identified socio-structural interventions, such as increased coverage of LLINs and IRS, greater access to quality education and improved quality of housing. Future studies that test the effect of different or joint effects of multiple mediators based on prospective designs are recommended to strengthen the evidence.

## Supplementary Information

Additional file 1.

## Data Availability

Data are available in a public, open-access repository. Data are freely available to the public on the DHS website.
