# Supplementary material for: The mediating role of behavioural and socio-structural factors on the association between household wealth and childhood malaria in Ghana
Source: Malar J. 2024 Dec 13;23:370. doi: 10.1186/s12936-024-05204-6 (PMC11645786; doi:10.1186/s12936-024-05204-6)
Supplement: Supplementary file 1 — Additional file 1. [file 12936_2024_5204_MOESM1_ESM.docx]

# **Supplement**

### **Supplementary file 1. Criteria for identifying mediator (The Baron and Kenny Approach, 1986)**

The causal diagram in Figure s1 captures the conceptualization of the role of a mediator variable. In this simple diagram, A represents an exposure variable, M denotes the mediating factor (mediator) and the outcome is denoted by Y.

M

Y

A

Fig s1. Simple DAG for the relation between exposure, mediator and outcome variables

**Baron and Kenny suggested four criteria for identifying a mediator but only two are generally accepted as correct**

**1. A change in levels of the exposure variable significantly affects the changes in the mediator (i.e., Path from A to M)**

In our study, change from low SEP (the lowest 40% of the scores) to high SEP (the top 20% of the scores) should affect changes in the mediator, the unadjusted estimates for the effect of SEP on mediators are shown below

1. **There is a significant relationship between the mediator and the outcome (i.e., Path from M to Y)**

We assessed crude associations between mediators and the outcome and presented the prevalence ratios and 95%CIs

**Table S1. Unadjusted associations between SEP and the mediators and also associations between the mediators and malaria**

| **Association between SEP and the mediators (unadjusted)** | | **Association between the mediators and malaria (unadjusted)** | |
| --- | --- | --- | --- |
|  | **PR (95% CI)** |  | **PR (95% CI)** |
| EA | 2.57 (2.28 – 2.89) | EA | 0.51 (0.44 – 0.60) |
| LLINs | 0.58 (0.51 – 0.67) | LLINs | 1.29 (1.11 – 1.49) |
| Housing quality | 1.97 (1.77 – 2.20) | Housing quality | 0.62 (0.54 – 0.71) |
| IRS | 0.59 (0.48 – 0.73) | IRS | 1.38 (1.17 – 1.63) |
| Healthcare- seeking in the last 6 months | 1.49 (1.27 – 1.74) | Healthcare- seeking in the last 6 months | 0.97 (0.83 – 1.13) |

**Abbreviations:** EA: Educational attainment; LLIN: Long lasting insecticide treated net; IRS: Indoor residual spraying; SEP: socioeconomic position. SEP was modelled as high, middle vs low; *results for middle are not presented*)

1. The third criterion is that a change in levels of exposure significantly changes the outcome. This has been critiqued by many scholars including Mackinnon 2008. A consensus has now been reached that the relationship between A and Y need not be statistically significant for M to be a mediator. The lack of significant association between X and Y could be due to suppression. Suppression happens when a mediating effect of a competing process has the opposite sign of the mediating effect of interest
2. When the previously defined paths are controlled, a previously significant relation between the exposure and outcome is no longer significant, with the strongest demonstration of mediation occurring when the path from the independent variable to the outcome variable is zero. This is no longer correct because there is possible partial mediation (i.e., there are potentially other mediators not considered). In any case, the change from significant to non-significant after adjusting for the mediator may be just a trivial change for example p-value change from 0.049 to 0.051. Whether this small change is indicative of mediation is questionable.

*This approach (Baron and Kennys) has fundamental flaws and we only use it as an explorative analysis. For mediation, we used modern methods - a counterfactual approach to mediation which we applied in this study.*
